# Neuromorphic Binarized Polariton Networks

**DOI:** 10.1021/acs.nanolett.0c04696

**Published:** 2021-02-26

**Authors:** Rafał Mirek, Andrzej Opala, Paolo Comaron, Magdalena Furman, Mateusz Król, Krzysztof Tyszka, Bartłomiej Seredyński, Dario Ballarini, Daniele Sanvitto, Timothy C. H. Liew, Wojciech Pacuski, Jan Suffczyński, Jacek Szczytko, Michał Matuszewski, Barbara Piętka

**Affiliations:** † Institute of Experimental Physics, Faculty of Physics, University of Warsaw, ul. Pasteura 5, PL-02-093 Warsaw, Poland; ‡ Institute of Physics, Polish Academy of Sciences, Aleja Lotników 32/46, PL-02-668 Warsaw, Poland; § CNR NANOTEC−Institute of Nanotechnology, Via Monteroni, 73100 Lecce, Italy; ∥ School of Physical and Mathematical Sciences, Nanyang Technological University, Singapore 637371

**Keywords:** exciton-polaritons, binary network, nonlinear optics, semiconductors, microcavities

## Abstract

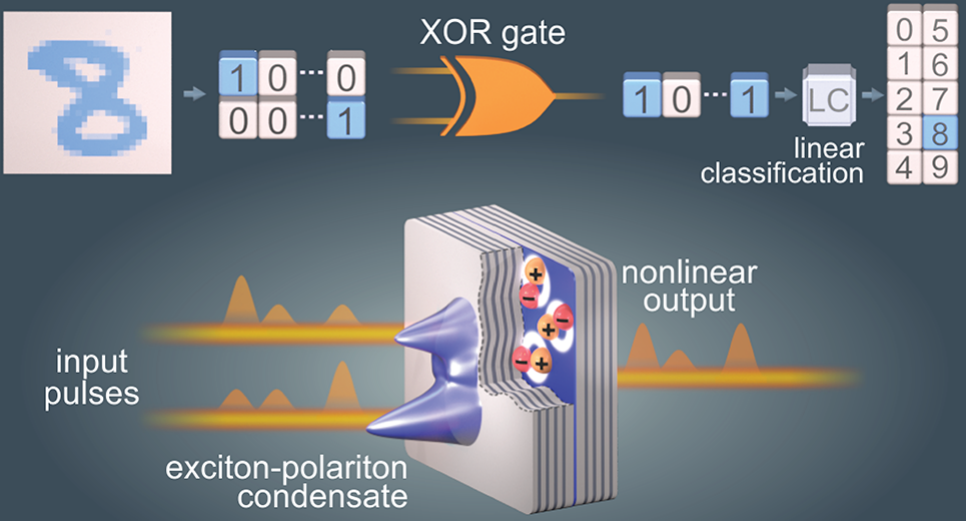

The rapid development of artificial neural networks and applied artificial intelligence has led to many applications. However, current software implementation of neural networks is severely limited in terms of performance and energy efficiency. It is believed that further progress requires the development of neuromorphic systems, in which hardware directly mimics the neuronal network structure of a human brain. Here, we propose theoretically and realize experimentally an optical network of nodes performing binary operations. The nonlinearity required for efficient computation is provided by semiconductor microcavities in the strong quantum light-matter coupling regime, which exhibit exciton–polariton interactions. We demonstrate the system performance against a pattern recognition task, obtaining accuracy on a par with state-of-the-art hardware implementations. Our work opens the way to ultrafast and energy-efficient neuromorphic systems taking advantage of ultrastrong optical nonlinearity of polaritons.

## Introduction

The human brain, despite consuming only about 15 W of power, is superior to the most advanced modern supercomputers in many practical tasks, such as object detection and classification. Artificial neural networks (ANNs) are an approach to data processing that mimics the operation of a biological network of neurons, allowing researchers to implement machine learning. Recent years have witnessed immense progress in ANN-based applied artificial intelligence, which has found many important applications in a growing diversity of fields, including medicine, logistics, finance, marketing, defense, agriculture, quantum science, geoscience, gaming, information technology, cybersecurity, language processing, robotics, and autonomous vehicles.^1,2^

As the amount of data continually grows, there is an urgent need to provide faster and more energy efficient systems. However, in comparison with the human brain, software simulations of neural networks are inefficient.^3^ In the von Neumann architecture, prevalent in conventional computers, the memory and processing units are physically separated, which results in a communication bottleneck. Moreover, the development of current semiconductor technology is bounded by the practical limit of Moore’s law and Amdahl’s law, which hinder the further increase of computational power through the decrease of system size or the increase of the number of processing units.^4^ These bounds are largely due to the limited energy efficiency of memory, communication channels, and processing units, which no longer improves exponentially as in the previous decades.^5^ Therefore, it is crucial to find an energy-efficient and powerful alternative for big data processing. Such a platform is required to realize a neuromorphic approach to neural networks, in which the massively parallel structure of the network is realized physically rather than simulated.^3^ In this context, photonic systems are natural candidates;^4,6−12^ but, most of the existing realizations were only able to perform basic machine learning tasks, and the advantage of optical system in terms of speed or energy efficiency has not been clearly demonstrated.

Recently, semiconductor microcavities in the quantum strong-coupling regime have emerged as a promising hardware platform for machine learning.^13,14^ Exciton-polaritons are quasiparticles resulting from the coupling between photons and excitons in this system.^15,16^ They exhibit properties of both light and matter. Electrostatic interactions of excitons lead to optical nonlinearity orders of magnitude stronger than in conventional optical media.^17,18^ The cavity photon lifetime results in a picosecond reaction time. The extremely low effective mass of polaritons allows for Bose–Einstein condensation^16,19^ recently realized at room temperature in organic and nonorganic materials,^20−22^ demonstrating strong nonlinear effects.^23,24^ Basic logic elements such as polariton switches, transistors, and gates have been realized.^18,25−30^ A system consisting of a polariton microcavity and an off-line classifier was demonstrated to outperform linear classification algorithms.^14^

To solve practical tasks of high complexity, a neural network has to perform a nonlinear transformation of input data into an effective higher-dimensional space. This allows for determining the result with a linear classification at the output layer.^31^ Recently, binarized neural networks, in which the activations or weights of connections are two-level and the neurons perform simple binary operations, have received much attention.^32,33^ Binarized networks are characterized by a greatly improved speed and energy efficiency, at the cost of a minimal reduction of inference accuracy.

Here, we propose theoretically and realize experimentally a binary network implemented in a polariton microcavity system. Importantly, the hardware of the network is composed of energy-passive optical elements only, such as resonators, beam splitters, and optical filters. We demonstrate that binarized neurons can operate in a fully all-optical mode, which allows for exploiting the intrinsic ultrashort time scales and high energy efficiency of photonics.^34^ The energy cost of a single binary operation is measured to be of the order of picojoules, which is comparable to the state-of-the-art electronic neuromorphic implementations, while the computation time scales are in the picosecond range. We demonstrate approximately 96% classification accuracy of handwritten digits from the Modified National Institute of Science and Technology (MNIST) data set, using a simple single-hidden-layer network in a noisy experimental environment.

## Results

### All-Optical XOR Logic Gate

**Figure 1:**
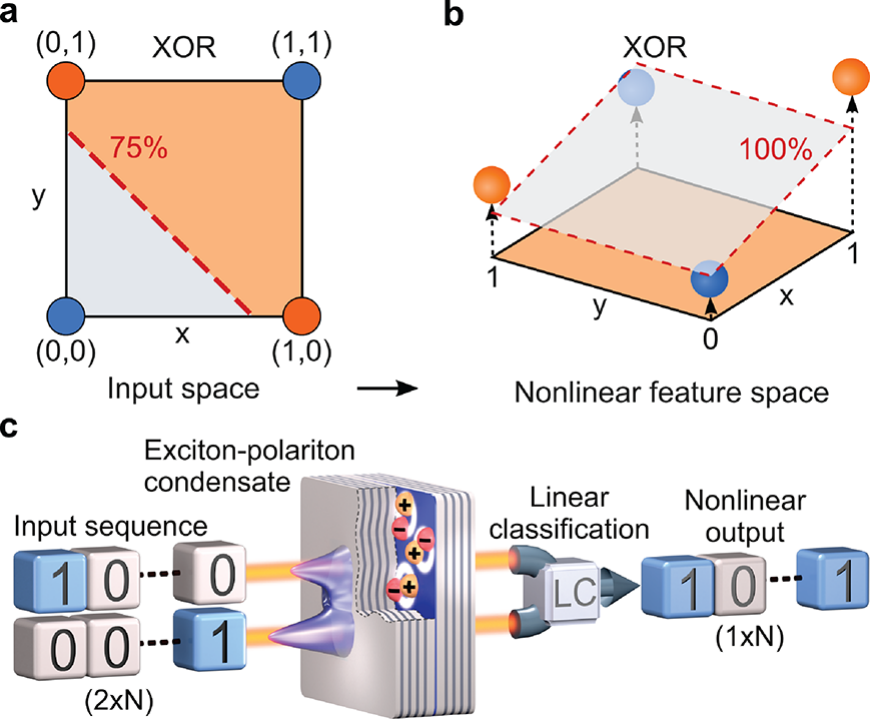
Nonlinear classification and experimental realization. (a) The XOR operation is a generic classification problem that is linearly inseparable in the space of inputs—there exists no straight line separating points corresponding to the “0” and “1” results marked with blue and orange circles, respectively. (b) An additional feature, represented by the *z* axis, which is a nonlinear function of inputs, allows for performing classification with a two-dimensional plane. (c) Experimental realization in an exciton–polariton system. A series of picosecond pulses encoding the inputs are incident on a semiconductor microcavity in the strong coupling regime, triggering a nonlinear response as a result of bosonic condensation. The emission is used to perform linear classification.

The first step in the implementation of a binarized network is the realization of its basic building block,^33^ a single XOR gate. The XOR task is a generic example of a problem not solvable using a perceptron or a linear classifier, see Figure 1a. Therefore, it is a benchmark of the capability to solve problems that require a nonlinear transformation. The principle of the implementation is depicted in Figure 1b. In addition to the inputs, which correspond to the two-dimensional *xy* plane in Figure 1b, a nonlinear feature (*z* axis) is provided by a micrometer-sized exciton–polariton condensate.

In our experiment, the microcavity consists of two CdTe-based Bragg mirrors, separated by an approximately 600 nm thick (Cd,Zn,Mg)Te layer. At the antinodes of the electromagnetic standing wave, six (Cd,Zn,Mn)Te quantum wells (QWs) are introduced for efficient coupling of QW excitons and the photonic modes (see the Supporting Information for more details).

We excite two spatially separated localized condensation sites, Figure 1c, with a series of nonresonant picosecond laser pulses, encoding the corresponding inputs with low (0) or high (1) pulse energy. The two sites are localized close to each other, with a 2 μm distance, which results in a Josephson junction type coupling.^35,36^ The light emitted from condensation sites is a nonlinear transformation of the inputs, directed to the linear classifier. The classifier is trained to distinguish “0” and “1” results by adjusting output weights, or the cut in the feature space (Figure 1b).

**Figure 2:**
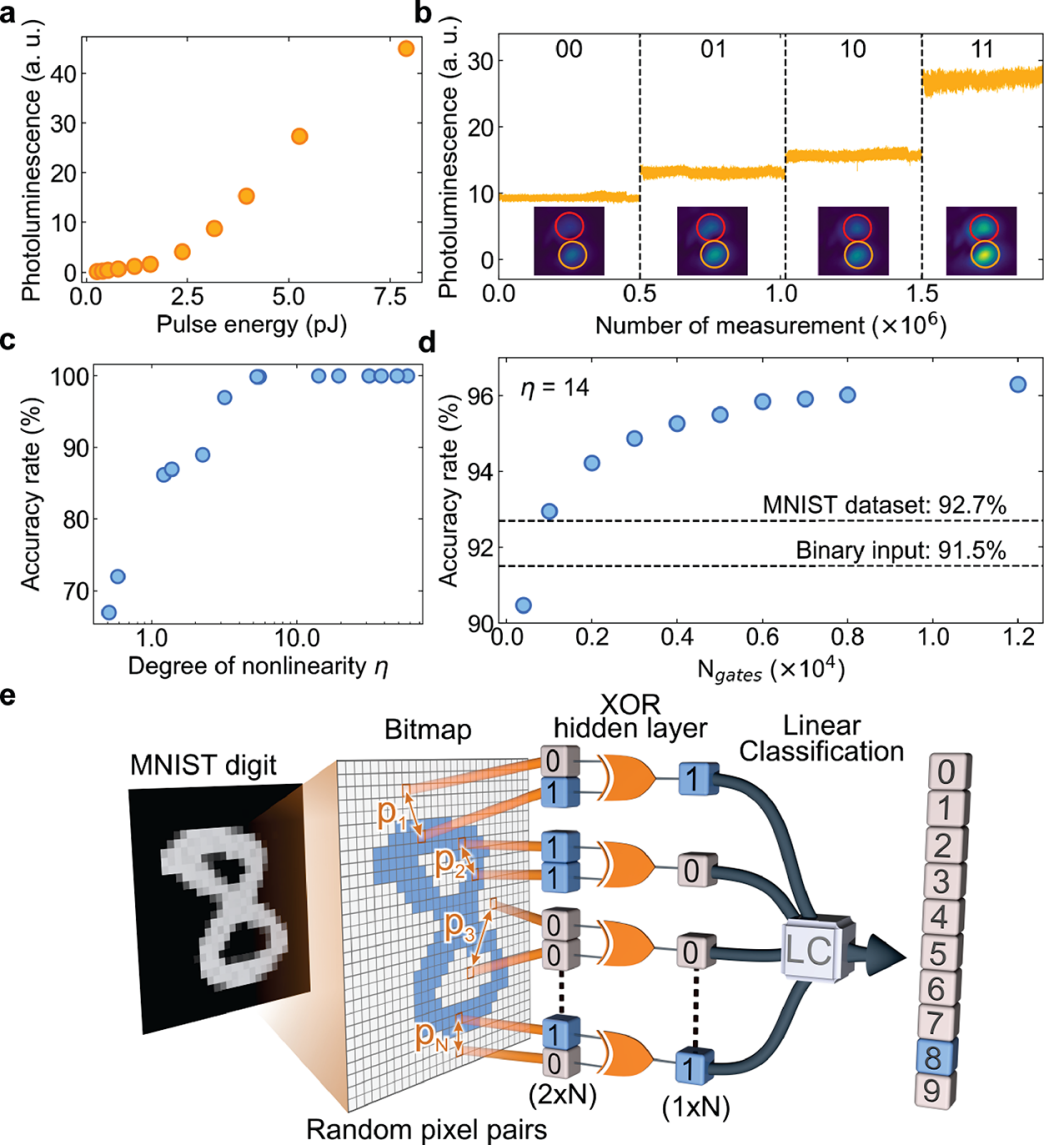
Optoelectronic machine learning. (a) The nonlinear dependence of the total emission intensity from the condensation site on the energy of two input pulses. (b) Emission in the four input configurations demonstrates nonlinearity. Insets show typical real-space emission observed on a CCD camera for each realization. The same color scale is preserved for each panel. Image size is of ∼7 μm × 7 μm. (c) Accuracy of the XOR gate as a function of the useful degree of nonlinearity η. (d) Accuracy of the MNIST handwritten digit prediction versus the number of XOR gates. Dashed lines show the benchmarks of software linear classification for the full and binarized MNIST input. (e) Conceptual scheme of the network with a single hidden layer of XOR gates.

Figure 2 shows the results obtained using an optoelectronic setup. The photoluminescence of a condensation site as a function of the combined pulse energy of the two inputs resembles the ReLU (rectified linear unit) activation function, see Figure 2a. Figure 2b shows the energy integrated output intensity from one of the sites for the four possible binary input combinations. The emission intensity from the two sites is converted to electronic signals by the camera and used to infer the result using linear classification. As demonstrated in Figure 2c, the accuracy (or the ratio of correct to total predictions) of the XOR gate depends on the degree of nonlinearity η (see the Supporting Information for the definition of η), and an almost perfect operation is obtained for η ≈ 5. Our system achieved perfect accuracy (no mistakes in several hundred thousand operations) due to the nonlinearity reaching η ≈ 50.

Having constructed the XOR unit, we build a binary network with a single hidden layer of several thousand (*N*_gates_) of XOR gates, see Figure 2e. We consider the handwritten digit recognition task using the MNIST data set, which consists of 60000 training samples and 10000 testing samples of 28 × 28 greyscale images.^37^ At the input, we convert each image into a black and white bitmap, and assign a random pair of pixels from the 28 × 28 image to each of the gates, see Figure 2e. The same pairs of pixel positions denoted by *p*_1_···*p*_*n*_ are assigned to the same gates 1···*n* for all digits. This allows us to detect nontrivial correlations between pixels even in the single-layer network. The above stage does not require any nonlinear operation and can be implemented all-optically, for example, using a three-dimensional laser-written waveguide array.^38^ Since the assignment is random and does not change during training, the structure of the network can be considered as a binary generalization of extreme learning machines.^39^ Deep networks with more complex structures can be implemented by cascading layers of XOR gates.^33^ To demonstrate the capability of the network, we use time multiplexing to realize all gates in the hidden layer. Logistic regression is used to determine the optimal classification hyperplane in the *N*_gates_-dimensional space (see the Supporting Information for details). The results are shown in Figure 2d, where we plot the accuracy of inference as a function of *N*_gates_. For around 10000 gates the accuracy reaches a plateau at the level of approximately 96%. This is comparable to or higher than that for the state-of-the-art neuromorphic implementations^9,14,40−42^ and is considerably higher than the accuracy of pure software linear classification of the grayscale MNIST data set (92.7%) or its binarized version (91.5%), obtained with logistic regression algorithm.

**Figure 3:**
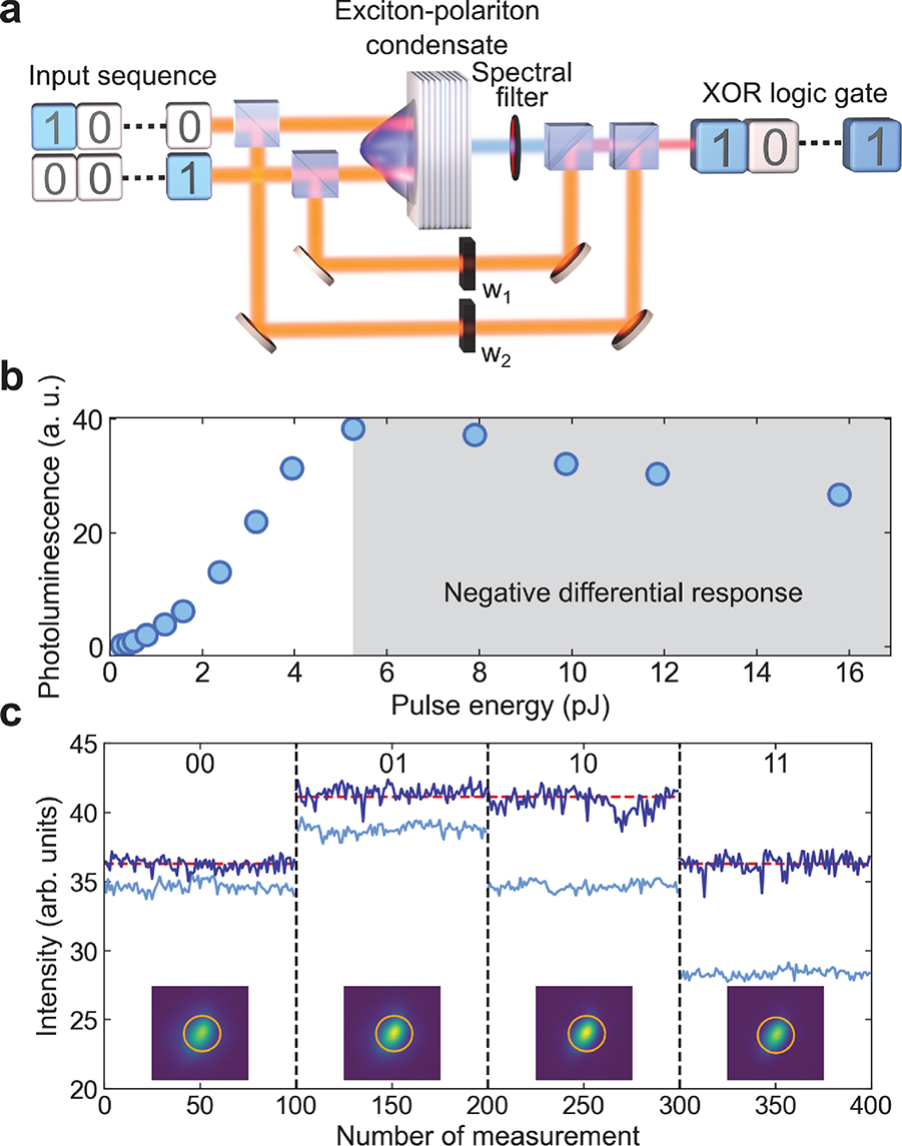
All-optical implementation of XOR gate. (a) Scheme of the experimental setup, in which the linear classification of Figure 1b is implemented with two auxiliary pulse paths controlled with neutral density filters, corresponding to weights *w*_1_ and *w*_2_. (b) Dependence of emission intensity on the energy of excitation pulses for equal pulse energy in both pulses. The spectral filter placed behind the sample allows for obtaining a negative differential response of the condensate emission. (c) Measured filtered emission intensity for all four combinations of inputs (blue) and the output intensity of the all-optical XOR gate (dark blue), which consists of the emission combined with the weighted inputs. Black dashed lines separate realizations of different inputs. Red dashed lines indicate the gate output intensity levels corresponding to results “0” and “1”. Insets show typical real-space emission observed on a CCD camera for each realization. The same color scale is preserved for each panel. Image size is of ∼6 μm × 6 μm.

Similar to the majority of photonic realizations,^7,8,40,43^ in the above scheme the linear classification is implemented electronically. This limits the speed and energy efficiency of the system. To solve this issue, we demonstrate that binarized neurons can operate in the all-optical configuration. Such a device is a photonic analog of neural network accelerators.^44−47^ In Figure 3a, we show the modified setup of the XOR gate, in which the linear classification is performed by optical elements only. The input pulses are directed at beam splitters, which create auxiliary optical paths bypassing the microcavity. In contrast to the previous scheme, a single condensation site is excited by both input pulses. The weights *w*_1_ and *w*_2_ of direct connections between the input and the output are implemented with neutral density filters, which reduce the pulse intensity in a controlled way. Since the emission from the condensate is always darker than the input pulses, the emission weight is set to unity. The emission from the condensate mixed with the two weighted auxiliary pulses constitutes the optical output of the gate. This intensity mixing effectively performs a simple three-component vector-matrix multiplication, which is necessary to perform the classification in the three-dimensional feature space (see the Supporting Information for details).

The nonlinear element has to exhibit a negative differential input–output dependence in a range of excitation powers, as shown in Figure 3b. As the filter weights *w*_1_ and *w*_2_ cannot be negative, the monotonically positive dependence would not lead to a useful gate (see the Supporting Information). We use a long-pass spectral filter placed behind the cavity to obtain the negative response shown in Figure 3b. In the “11” input configuration the polariton–polariton interactions shift the emission to higher frequencies, which are blocked by the filter. This method allows for obtaining the well-defined “0” and “1” output levels, which are consistent for all input configurations, see Figure 3c. The noise of the output results mostly from the limited stability of our laser.

To estimate the energy efficiency we determine the input pulse energy required for a single gate operation. The power of input pulses in the “1” state was measured using a power meter at the entrance to the microcavity to be 1.2 mW at 76 MHz repetition rate, which gives approximately 16 pJ pulse energy per gate operation, while the energy of auxiliary pulses was much lower. The approximate cost is around 16 pJ per synaptic operation, comparable to the state-of the-art neuromorphic electronic implementation.^3^

### Discussion

The radical change of the paradigm of computation allows us to propose an optical system that can be realized with currently available optical elements. In particular, the system does not require a separate memory unit, as all information is carried by photons propagating through the network. We emphasize that despite the binary structure of the network, which is based on XOR gates, we go beyond the traditional digital computer architecture. Our approach reveals the potential of semiconductor microcavity systems as a platform for energy efficient information processing.

## Supporting Information Available

Sample, polariton spectra, experimental setup, degree of useful nonlinearity, optoelectronic machine learning, the XOR classification task, all-optical XOR operation ultrafast XOR gate, and teaching the network for the MNIST handwritten digits classification task (PDF)
