# Supplementary material for: Neuromorphic Binarized Polariton Networks
Source: Nano Lett. 2021 Feb 26;21(9):3715–20. doi: 10.1021/acs.nanolett.0c04696 (PMC8155323; doi:10.1021/acs.nanolett.0c04696)
Supplement: Supplementary file 1 — nl0c04696_si_001.pdf [file nl0c04696_si_001.pdf]

Neuromorphic binarized polariton networks

Supporting Information

Rafał Mirek, Andrzej Opala, Paolo Comaron, Magdalena Furman, Mateusz Król, Krzysztof Tyszka, Bartłomiej Seredyński, Dario Ballarini, Daniele Sanvitto, Timothy C. H. Liew, Wojciech Pacuski, Jan Suffczyński, Jacek Szczytko, Michał Matuszewski,* and Barbara Piętka*

E-mail: mmatu@ifpan.edu.pl; barbara.pietka@fuw.edu.pl

Sample

The investigated system is a semiconductor heterostructure consisting of two distributed Bragg reflectors with 16 and 19 alternating (Cd,Zn,Mg)Te/(Cd,Mg)Te layers, respectively, separated by an approximately 600 nm thick (Cd,Zn,Mg)Te layer, forming a microcavity of quality factor 300. The microcavity contains three pairs of quantum wells of 20 nm with a small concentration (about 0.5%) of manganese ions each. The structure was grown on a (100)-oriented GaAs substrate by molecular beam epitaxy. The detailed scheme of the structure is presented in Figure 1.

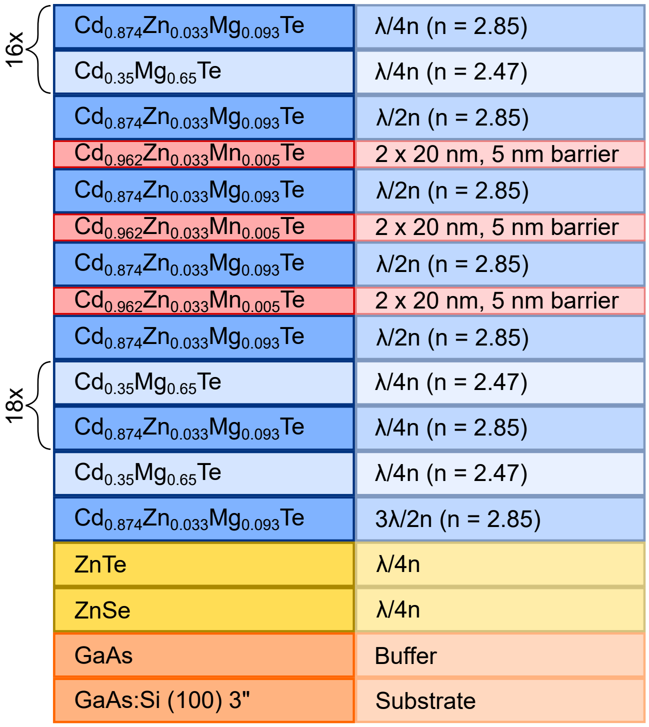

Figure 1: The structure of the sample: six 20 nm-wide QWs (red) containing manganese ions placed between 19 layers (bottom) and 16 layers (top) of non-magnetic distributed Bragg reflectors (alternating light blue and blue layers).

Polariton spectra

Figure 2 illustrates the typical dispersion observed for a negative exciton-photon detuning. The fitting of the two-level model to the reflectivity map reveals the energy of the uncoupled exciton (1.614 eV), uncoupled photon (1.607 eV at zero wavevector) and Rabi energy (10.4 meV). We observe the lower polariton with a characteristic bottleneck at large momenta in a photoluminescence map. The upper polariton is very weakly occupied due to efficient relaxation to the lower polariton state. In Figure 3 we show the polariton spectra for a posi-

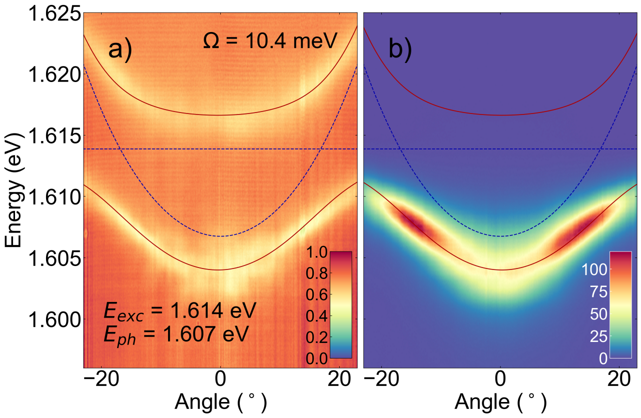

Figure 2: Angle-resolved reflectivity (a) and photoluminescence (b) maps for negatively detuned position on the sample. Red curves indicate the energies of polariton branches. The energies of the bare exciton and photon obtained from the fitting to the reflectivity map are marked by the blue dashed curves.

tion on the sample where the all-optical XOR gate was realised. We take advantage from the fact that the nonlinear effects can be enhanced in localised states[1–3] and we focus in the trap formed due to the natural photonic potential fluctuations. For low excitation power we observe the lower polariton branch with localised states marked by the black lines. In the realization of an XOR gate we use a spectral filter cutting off the emission above the first localised state. It is worth mentioning that the filter is not perfectly sharp and decreases the intensity of the lowest state. Above condensation threshold additional states appear that are cut-off by the spectral filter. In Figure 4 we present the energy blue-shift and linewidth (FWHM) of the lowest energy mode for increasing pulse energy. We observe the increase of the mode energy due to polariton-polariton interactions up to 2.5 meV. The FWHM decreases from 2 meV to below 0.5 meV as a consequence of the condensation process.

Experimental setup

The experimental setup is presented in Figure 5. A pulsed picosecond laser (Mira 900) with 76 MHz repetition rate non-resonantly

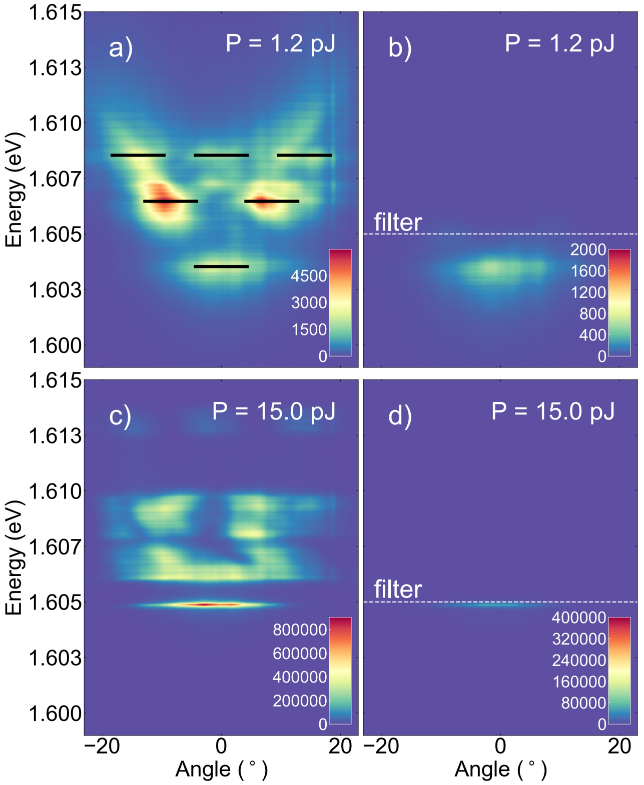

Figure 3: Angle-resolved photoluminescence maps of negatively detuned (-7 meV) exciton-polaritons below (a,b) and above threshold (c,d). Right panels show photoluminescence maps after the long pass filter (780 nm) rotated at a small angle giving a cut-off at 1.605 eV. Discrete energy states observed due to localization in photonic potential are marked with black lines.

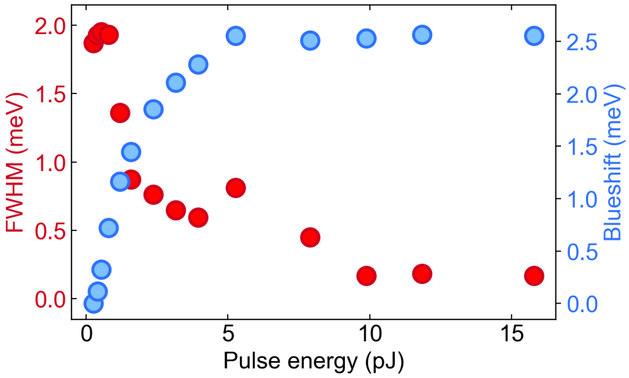

Figure 4: The energy shift and linewidth (full width at half maximum, FWHM) of the lowest energy mode for increased energy in the excitation pulse.

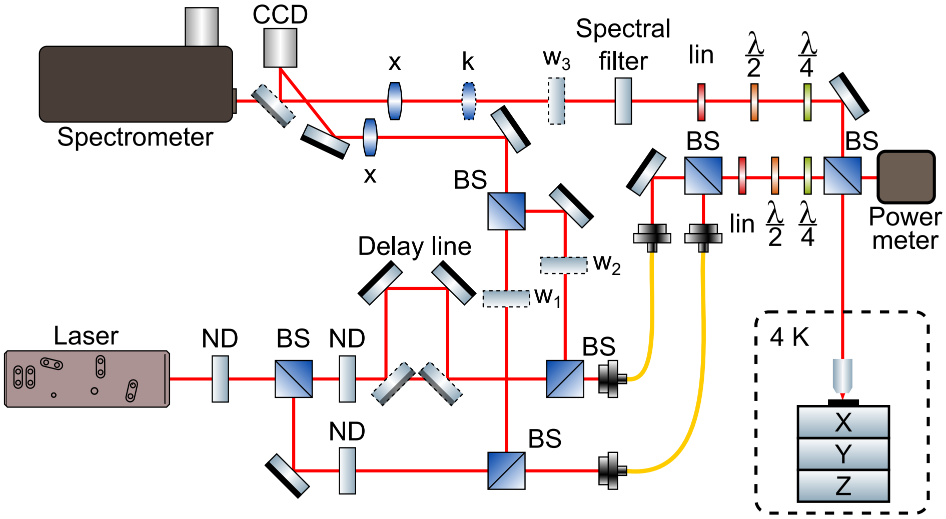

Figure 5: Experimental setup for the realization of all-optical XOR gate.

excites polariton condensates with σ^+ polarised light at the energy $E_{exc} = 1.724\,\mathrm{eV}$ ($\lambda_{exc} = 719\,\mathrm{nm}$). The laser output is split into two pulses and then coupled into fibers. The time delay between the pulses can be tuned by changing the position of the mirrors on a delay line from 0 ps to 666 ps. To avoid interference effects at exactly zero delay time, the smallest delay between the pulses is set to 14 ps for an experiment where both laser pulses excite a single condensation site. Neutral-density filters placed before the fiber couplers independently control the excitation power of each pulse.

The sample is placed inside an optical cryostat at the temperature of liquid helium. Laser pulses are focused on the sample by objective with high numerical aperture (of 0.68). The position of each laser spot can be tuned separately and the distance is set to $2\,\mu m$ for the two coupled condensates scheme, or they completely overlap for the single condensate excitation scheme. Emission from the sample is collected by the same objective. Due to the limited detection efficiency of our CCD camera, the signal is averaged over 200 ms. As it was shown experimentally, it is possible to perform single-pulse measurements on exciton-polariton condensates.[4]

Degree of useful nonlinearity

The nonlinearity of the emission can be quantified as the combination of the average intensities, $\Delta I = \langle I_{00}\rangle + \langle I_{11}\rangle - \langle I_{01}\rangle - \langle I_{10}\rangle$. For a linear device, this quantity is equal to zero. We define the useful nonlinearity as the ratio of the nonlinear response to the amplitude of noise present in the system: $\eta = \Delta I/\sqrt{V}$ with $V = V_{00} + V_{01} + V_{10} + V_{11}$, where V_{ii} is the variance of intensity in the (i, j) input configuration, $V_{ij} = \left\langle (I_{ij} - \langle I_{ij}\rangle)^2 \right\rangle$. Variance is caused mainly by the instability of the laser intensity while fluctuations of the condensate intensity are minor effects. If the degree of useful nonlinearity is high (nonlinear effects in polaritons are more significant than the noise), there is very little probability that the plane separating the two regions in the feature space of Figure 1b in the main text will give an incorrect prediction due to the experimental noise. Note that in Figure 2c in main text, we only plot the nonlinearity of one of the two features (emission from one of the two spots), which is characterized by a higher degree of nonlinearity, since the linear classification is able to assign a higher weight to the more useful feature.

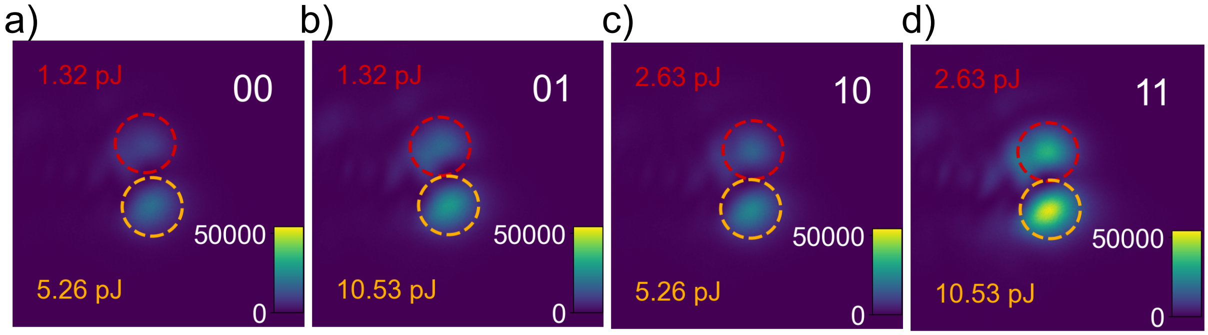

Figure 6: Real space emission of two spatially-separated polariton condensates excited with (a) low/low, (b) low/high, (c) high/low and (d) high/high excitation powers. Color code corresponds to the emission intensity. Left annotations in the panels describe the energy of the pulse applied to the first (marked with red circle) and second (marked with yellow circle) condensate. Image size is of $10\,\mu m \times 10\,\mu m$.

Opto-electronic machine learning

In order to obtain a nonlinear logic gate we focus two laser pulses on the two condensation sites separated in space by about 2 μm. Both laser pulses are exciting the sample at the same time (with the delay time set to 0 ps). The four logic states are obtained by changing pulse energy focused on each condensation site. Emission from the sample is collected by the CCD camera (see Figure 6). In our experiment the condensates are additionally weakly laterally localized in photonic potential traps. This assures evanescent coupling between the condensates.[5,6] In general, this localization is not an important condition and the same results can be obtained for any optically created polariton network as in Refs.[7–9]

The XOR classification task

The XOR gate can be realized in the three dimensional feature space composed of the two inputs, x–y plane, and a single additional feature, represented by the z axis, as shown in Figure 1b in the main text. In principle this feature can correspond to any physical quantity with an arbitrary small nonlinearity. In the three dimensional space it is always possible to find a plane which contains three arbitrary points, and the plane will contain the fourth point only if they are linearly dependent. If the points are not linearly dependent, the plane can be adjusted in such a way that the point corresponding to the same class as the fourth point is on the same side of the plane, while the two points corresponding to the different class are on the other side. This can be done by infinitesimally small shifts of the plane, hence the nonlinearity can be arbitrarily small. In a real system, the output is noisy for all four input configurations of the gate. In this case we need to separate finite sized regions rather than infinitely small points. Consequently, the nonlinearity has to be larger than the noise present in the system.

The result of the XOR operation can be predicted from the sign of the expression defining the plane $w_1x + w_2y + w_3z = b$, where w_1 and w_2 are the input weights, w_3 is the weight of the nonlinear output feature and b is the bias. We determine the weights using the linear regression algorithm with no regularization. We can also choose to set the weight w_3 to unity (for $w_3 = 0$, the problem is equivalent to the two-dimensional case) and the other parameters can be appropriately rescaled. In an ideal case, with no noise in the system, it is sufficient to measure each of the four input combinations only once to determine optimal weights.

Importantly, in our hardware implementation all weights have to be positive as they are implemented with optical filters. It would not be possible to find three positive weights if the nonlinear feature was a monotonously growing

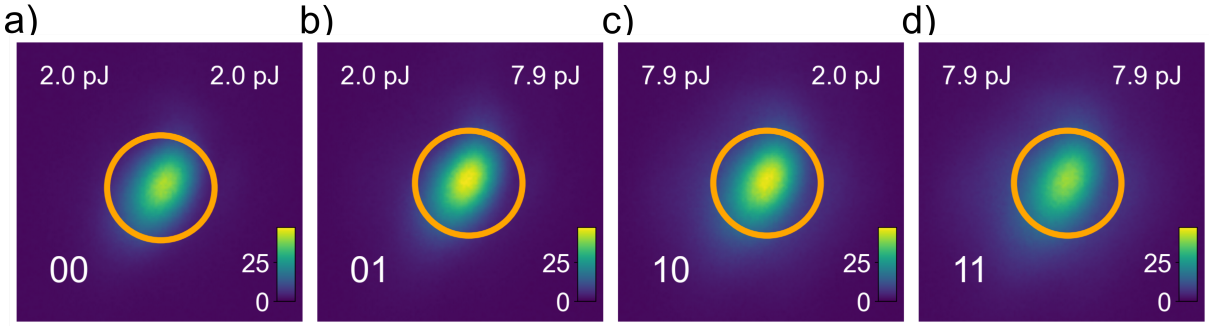

Figure 7: Real space emission of polariton condensate excited with (a) low/low, (b) low/high, (c) high/low and (d) high/high excitation powers. Color code corresponds to the emission intensity. Top annotations in the panels describe the energy of the first (left) and second (right) pulse applied to the condensate. Delay time between the pulses was 17 ps to avoid interference effects between the laser pulses. The response of the system was different in the cases when the pulse of high energy was the first one ("10" configuration) and when the pulse of low energy was the first one ("01" configuration). Image size is of $6\,\mu m \times 6\,\mu m$.

function of inputs. To demonstrate this, let us consider the output of the gate given by $h = w_1 x + w_2 y + w_3 z$. For positive weights, the "11" configuration would always give a higher output value than both "10" and "01" configurations, while the "00" configuration would result in a lower value. It is then important that the nonlinear element provides a negative differential response $z(x, y)$ at least in the range of excitation energies that is used to encode the inputs.

All-optical XOR operation

In this experiment two laser pulses are focused on the same position on the sample. The delay time between the pulses was set to 14 ps to avoid unnecessary interference effects between the laser pulses. The all-optical linear classification of the output data is realized through mixing of the input and output signals. First, before coupling the laser pulses into the fibers, we split each of them and apply weights w_1 and w_2 through neutral density filters. Second, we overlap these additional laser pulses with the output signal from the sample directly on the detector. In this configuration we are able to control and measure both inputs and outputs. Long-pass spectral filter cutting off the emission above 1.605 eV ensures negative differential response of the system. Above a certain

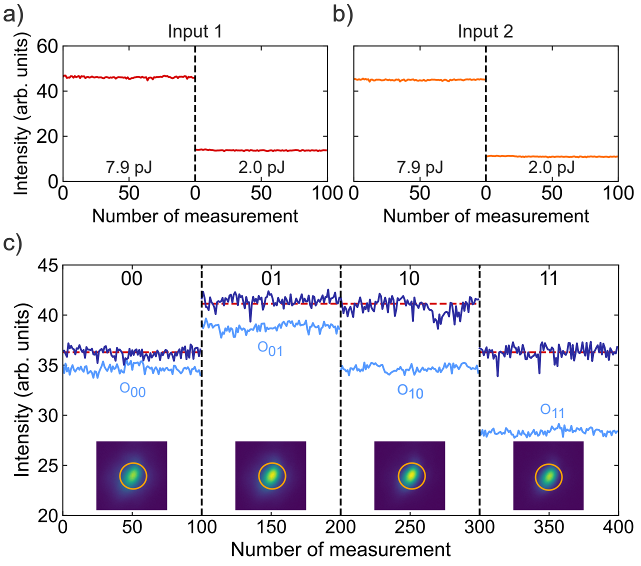

Figure 8: Three dimensional feature space realized for XOR gate operation. Intensity of the (a) input 1 and (b) input 2 signals creating x–y plane (depicted in Figure 1b of the main text). c) Measured filtered emission intensity for all four combinations of inputs (blue) and the output intensity of all-optical XOR gate (dark blue), which consists of the emission combined with the weighted inputs (w_1=0.16 and w_2=0.03).

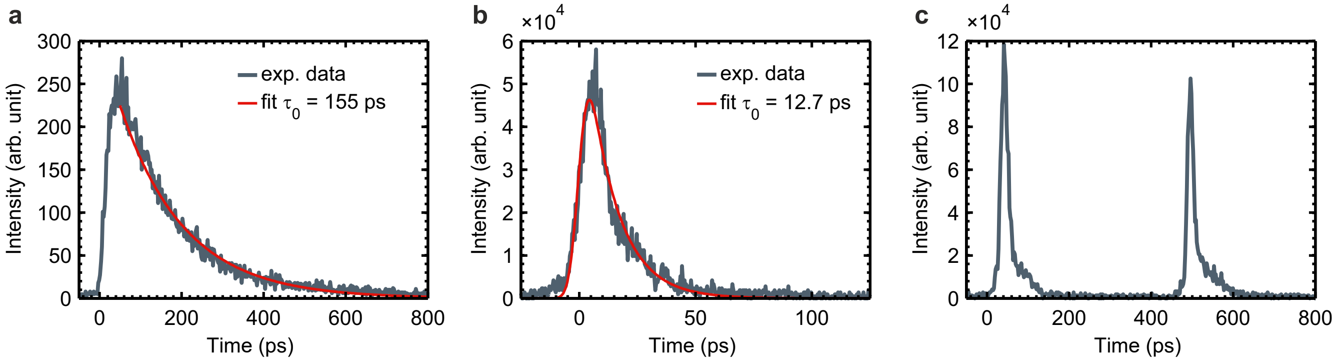

Figure 9: **a** Decay of polariton reservoir for excitation 50 μW, below the condensation threshold. **b** Decay of polariton condensate excited with 160 μW. Fit is a convolution of excitation gaussian pulse with FWHM = 7.7 ps and exponential decay with time τ_0. **c** Two polariton condensates excited by two 100 μW beams delayed by 460 ps.

energy of the pulses blue shifted emission is getting weaker in intensity. In the experiment, the energy of both pulses is changed independently to realize different logic states. Total emission integrated in time is collected by the CCD camera. Figure 7 shows four logic realizations of all-optical XOR observed in photoluminescence.

In Figure 8 we show practical realization of the XOR classification task. We measure on the CCD camera intensity of the first (a) and the second (b) input in the low and high state. Then, we measure emission from the sample for all four input configurations (light blue curve on (c)). The PL signal is integrated in energy and spatially from the region enclosed in the circles marked in the insets. Finally, we can find weights allowing for XOR gate operation. In this case obtained weights were: w_1=0.16 and w_2=0.03, and we set w_3=1. The resulting signal (dark blue) is the sum of the weighted inputs and the emission.

Ultra-fast XOR gate

To confirm the ultra-fast operation of our XOR logic gate we used a streak camera to detect the gate output in real-time with high temporal resolution. First, we observe that in response to a single laser beam with mean power (50 μW) below the condensation threshold the emitted output intensity decays with time $\tau = 155$ ps (Figure 9a). This value corresponds to the lifetime of the polariton reservoir. In this case, the photoluminescence is still visible even above 400 ps. In contrast, for the input pulse with mean power above the threshold, the emission decay is faster and corresponds to the shorter lifetime, of about 13 ps, of polariton condensate (Figure 9b). The emission vanishes after 100 ps. To demonstrate the ultra-fast operation we used two 100 μW laser pulses delayed in time by 460 ps and incident on the same condensation site. We observed two response pulses, which exhibit the same intensity and decay time and correspond to subsequent emission from two independent condensates, as shown in Figure 9c. Based on these results, we can confirm that a single XOR operation can be completed in less than 500 ps for non-resonant excitation.

Teaching the network for the MNIST handwritten digits classification task

In the handwritten digits recognition task, we use a supervised learning method based on softmax regression. The softmax regression is a generalization of logistic regression in the multiple classes case. In the MNIST classification problem, softmax regression returns the separate probabilities for each digits class (0–9). To perform classification we use a simple linear mapping method: $f(\mathbf{x}_i, \mathbf{W}, \mathbf{b}) = \mathbf{x}_i\mathbf{W} + \mathbf{b}$, where $\mathbf{x}_i$ is a vector of input states, $\mathbf{W}$ is the weights matrix and $\mathbf{b}$ is a bias vector. Before

the training process, the weights matrix is randomly distributed. The weights are obtained by training the system, minimizing the loss function given by the cross-entropy. The minimization of the loss function is executed using Adam optimizer. The software algorithms are implemented with Tensorflow[10] platform on a standard PC with GeForce RTX 2070 Graphics Processing Unit.

References

(1) Verger, A.; Ciuti, C.; Carusotto, I. Polariton quantum blockade in a photonic dot. *Phys. Rev. B* **2006**, *73*, 193306.

(2) Abbarchi, M.; Amo, A.; Sala, V. G.; Solnyshkov, D. D.; Flayac, H.; Ferrier, L.; Sagnes, I.; Galopin, E.; Lemaître, A.; Malpuech, G.; Bloch, J. Macroscopic quantum self-trapping and Josephson oscillations of exciton polaritons. *Nature Physics* **2013**, *9*, 275–279.

(3) Rahimzadeh Kalaleh Rodriguez, S.; Amo, A.; Carusotto, I.; Sagnes, I.; Le Gratiet, L.; Galopin, E.; Lemaitre, A.; Bloch, J. Nonlinear Polariton Localization in Strongly Coupled Driven-Dissipative Microcavities. *ACS Photonics* **2018**, *5*, 95–99.

(4) Estrecho, E.; Gao, T.; Bobrovska, N.; Fraser, M. D.; Steger, M.; Pfeiffer, L.; West, K.; Liew, T. C. H.; Matuszewski, M.; Snoke, D. W.; Truscott, A. G.; Ostrovskaya, E. A. Single-shot condensation of exciton polaritons and the hole burning effect. *Nature Communications* **2018**, *9*, 2944.

(5) Kaitouni, R. I.; El Daïf, O.; Baas, A.; Richard, M.; Paraiso, T.; Lugan, P.; Guillet, T.; Morier-Genoud, F.; Ganière, J. D.; Staehli, J. L.; Savona, V.; Deveaud, B. Engineering the spatial confinement of exciton polaritons in semiconductors. *Phys. Rev. B* **2006**, *74*, 155311.

(6) Amo, A.; Bloch, J. Exciton-polaritons in lattices: A non-linear photonic simulator. *Comptes Rendus Physique* **2016**, *17*, 934–945.

(7) Ballarini, D.; Gianfrate, A.; Panico, R.; Opala, A.; Ghosh, S.; Dominici, L.; Ardizzone, V.; De Giorgi, M.; Lerario, G.; Gigli, G.; Liew, T. C. H.; Matuszewski, M.; Sanvitto, D. Polaritonic Neuromorphic Computing Outperforms Linear Classifiers. *Nano Letters* **2020**, *20*, 3506–3512.

(8) Töpfer, J. D.; Sigurdsson, H.; Pickup, L.; Lagoudakis, P. G. Time-delay polaritonics. *Communications Physics* **2020**, *3*, 2.

(9) Ohadi, H.; Ramsay, A. J.; Sigurdsson, H.; del Valle-Inclan Redondo, Y.; Tsintzos, S. I.; Hatzopoulos, Z.; Liew, T. C. H.; Shelykh, I. A.; Rubo, Y. G.; Savvidis, P. G.; Baumberg, J. J. Spin Order and Phase Transitions in Chains of Polariton Condensates. *Phys. Rev. Lett.* **2017**, *119*, 067401.

(10) Abadi, M. et al. TensorFlow: A System for Large-Scale Machine Learning. 12th USENIX Symposium on Operating Systems Design and Implementation (OSDI 16). Savannah, GA, 2016; pp 265–283.
